# Projected Normal Distribution: Moment Approximations and Generalizations

**Published:** 2025-06-20

**Authors:** Daniel Herrera-Esposito, Johannes Burge

**Affiliations:** 1 Department of Psychology, University of Pennsylvania, Hamilton Walk, Philadelphia, 19104, Pennsylvania, United States.

**Keywords:** Projected normal distribution, Angular Gaussian, Directional statistics, Moments approximation, Quadratic forms, Divisive normalization

## Abstract

The projected normal distribution, also known as the angular Gaussian distribution, is obtained by dividing a multivariate normal random variable x by its norm $\sqrt{x^{T}x}$. The resulting random variable follows a distribution on the unit sphere. No closed-form formulas for the moments of the projected normal distribution are known, which can limit its use in some applications. In this work, we derive analytic approximations to the first and second moments of the projected normal distribution using Taylor expansions and using results from the theory of quadratic forms of Gaussian random variables. Then, motivated by applications in systems neuroscience, we present generalizations of the projected normal distribution that divide the variable x by a denominator of the form $\sqrt{x^{T}Bx+c}$, where B is a symmetric positive definite matrix and c is a non-negative number. We derive moment approximations as well as the density function for these other projected distributions. We show that the moments approximations are accurate for a wide range of dimensionalities and distribution parameters. Furthermore, we show that the moments approximations can be used to fit these distributions to data through moment matching. These moment matching methods should be useful for analyzing data across a range of applications where the projected normal distribution is used, and for applying the projected normal distribution and its generalizations to model data in neuroscience.

## 1 Introduction

The projected normal distribution, also known as the angular Gaussian distribution, is a probability distribution on the unit sphere ${𝒮}^{n-1}$. A random variable $y\in {𝒮}^{n-1}$ that is distributed according to the projected normal distribution y~𝒫𝒩(μ,Σ) is obtained by radially projecting a random vector x~𝒩(μ,Σ) onto ${𝒮}^{n-1}$ as y=x/‖x‖^1^. Some appealing properties of the projected normal distribution are that it is simple to sample from, that it can be easily generalized to arbitrary dimensions, and that variants of the distribution can be readily obtained by imposing constraints on the parameters of the variable x (Paine et al. 2018).

However, the projected normal distribution has seen rare use in the classical literature on directional statistics. The paucity of its use is likely due to its relatively complicated density, the fact that it has a larger number of parameters compared to other angular distributions (e.g. the Kent distribution), and the lack of closed-form formulas for its moments (Wang and Gelfand 2013; Paine et al. 2018). Nonetheless, interest in the projected normal distribution is growing, due to its intuitive simplicity, recently described variants of the distribution with fewer parameters (Paine et al. 2018), and computational advances in parameterization and sampling methods (Wang and Gelfand 2013; Hernandez-Stumpfhauser et al. 2017; Yu and Huang 2024).

Recent work in physics, biology, and in Deep Learning, has used the projected normal distribution to model data on the circle, the sphere, and the hypersphere (Paine et al. 2018; Hernandez-Stumpfhauser et al. 2017; Maruotti 2016; Yu and Huang 2024; Michel et al. 2024). It has also recently been proposed that the projected normal distribution is a natural choice to model the effects of divisive normalization on response variability in neural populations (Herrera-Esposito and Burge 2024). Divisive normalization is a model describing the sublinear relation between the inputs and outputs of a neural population (Carandini and Heeger 2012). A simple version of the model can be given by y=x/‖x‖, where x is a vector of inputs to a neural population, and y is the vector of the neural responses or outputs. More flexible divisive normalization schemes use denominators of the form $\sqrt{x^{T}Bx+c}$, where B is a symmetric positive definite matrix and c is a non-negative constant (Carandini and Heeger 2012; Coen-Cagli et al. 2012; Coen-Cagli and Schwartz 2013).

One particular question of interest in systems neuroscience is how divisive normalization interacts with noise in the neural inputs to shape the response properties of neural population responses, especially the mean and the noise covariance of the neural responses (Goris et al. 2014; Verhoef and Maunsell 2017; Coen-Cagli and Solomon 2019; Weiss et al. 2023; Goris et al. 2024). However, models in computational neuroscience that incorporate divisive normalization have mostly been used to account for mean neural responses. Response (co)variance is most often added as an independent source of noise (but see Coen-Cagli and Schwartz (2013); Goris et al. (2014); Coen-Cagli and Solomon (2019); Weiss et al. (2023)). There is a need for statistical models that can describe the joint effects of input noise and divisive normalization on neural population responses. Notably, by letting the neural population inputs x be a random variable with a multivariate normal distribution–a common noise assumption in models of neural systems (Kohn et al. 2016; Coen-Cagli and Solomon 2019; Goris et al. 2024)–the projected normal distribution and some generalized versions of it become a natural choice for modeling how input neural noise interacts with divisive normalization. One limitation of the projected normal distribution for this and other applications, however, is that no formulas for the first and second moments of the projected normal distribution are known.

**Fig. 1:**
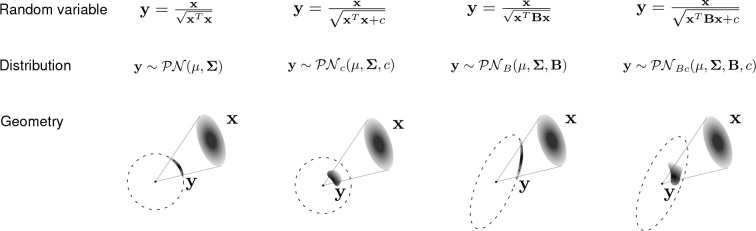
Geometry of the projected normal for the 2D case, and the generalizations considered here. Each column shows one of the four distributions considered in this work. The first row show the formula corresponding to each version of the corresponding random variable y. Each distribution is obtained by dividing the Gaussian distributed random variable x~𝒩(μ,Σ) by a different denominator. The second row shows the notation used to refer to each version of the random variable y. The third row shows the geometry of each projected variable y. The first column shows the random variable y with a projected normal distribution, which projects x onto the unit sphere ${𝒮}^{n-1}$. The second column shows the random variable y with an added constant c in the denominator, which projects x into the interior of the unit sphere. The third column shows the random variable y with a symmetric positive definite matrix B in the denominator, which projects x onto the surface of an ellipsoid. The fourth column shows the random variable y with a symmetric positive definite matrix B and an added constant c in the denominator, which projects x into the interior of an ellipsoid.

Here, we derive analytic approximations to the first and second moments of the projected normal distribution 𝒫𝒩(μ,Σ). Then, motivated by applications in systems neuroscience, we also derive moment approximations and density functions for generalizations of the projected normal distribution having normalizing denominators of the form $\sqrt{x^{T}Bx+c}$, where B is a symmetric positive definite matrix and c is a non-negative constant (Figure 1). In all cases, we show that the moment approximations are accurate, and that they can be used to fit the projected normal distribution and its generalizations to data via moment matching. This novel fitting method and the generalizations of the projected normal distribution can be useful for modeling data in a range of applications, including systems neuroscience (Carandini and Heeger 2012; Goris et al. 2014; Coen-Cagli and Solomon 2019; Weiss et al. 2023; Goris et al. 2024).

## 2 The projected normal distribution 𝒫𝒩(μ,Σ)

### Code

All the formulas and fitting procedures presented in this work are implemented as a Python package named ‘projnormal’, available at https://github.com/dherrera1911/projnormal.

### Notation

Plain lower case letters denote scalars (a), bold lower case letters denote vectors (a), and bold upper case letters denote matrices (A). Greek letters denote moments of distributions. An overhead bar $\overset{-}{\cdot }$ is used to denote the mean of a random variable, var(·) to denote the variance, and cov(·,·) to denote the covariance between two random variables. A distribution parameter with a hat (e.g. $\hat{\Sigma }$) denotes a model parameter fitted to data, rather than the true underlying parameter of the distribution. A moment of the distribution with a tilde (e.g. $\tilde{\gamma }$) denotes a moment approximated using the formulas derived in this work.

### 2.1 Probability density function of 𝒫𝒩(μ,Σ)

The density function of $y=x/\|x\|\in {𝒮}^{n-1}$, where x~𝒩(μ,Σ), is given by (Pukkila and Radhakrishna Rao 1988; Paine et al. 2018):

(1) $$p(y;\mu ,\Sigma )=\frac{e^{\frac{1}{2}\left(q_{2}^{2}/q_{3}-q_{1}\right)}}{det(2\pi \Sigma {)}^{\frac{1}{2}}q_{3}^{\frac{n}{2}}}{ℳ}_{n-1}\left(\frac{q_{2}}{q_{3}^{2}}\right)$$

where $q_{1}=\mu^{T}\Sigma^{-1}\mu$, $q_{2}=\mu^{T}\Sigma^{-1}y$, $q_{3}=y^{T}\Sigma^{-1}y$, and ${ℳ}_{n-1}(\alpha )$ is a function computed recursively as

$${ℳ}_{n+1}(\alpha )=\alpha {ℳ}_{n}(\alpha )+n{ℳ}_{n-1}(\alpha )$$

with the initial values ${ℳ}_{0}=\Phi (\alpha )$ and ${ℳ}_{1}=\alpha \Phi (\alpha )+\phi (\alpha )$. ϕ(⋅) and Φ(⋅) are the standard normal probability density function and cumulative probability function, respectively. This formula has been used to fit the projected normal parameters μ and Σ to data with maximum likelihood through iterative optimization (Paine et al. 2018).

### 2.2 Approximation to the moments of 𝒫𝒩(μ,Σ)

To approximate the first and second moments of y~𝒫𝒩(μ,Σ) we use Taylor expansions, and formulas for the moments of quadratic forms in Gaussian random vectors. We denote the first moment and the covariance of y as γ=E[y] and $\Psi =E[(y-\gamma )(y-\gamma {)}^{T}]$, respectively.

#### 2.2.1 First moment approximation

To approximate the element $\gamma_{i}=E\left[y_{i}\right]$ of the first moment, we define the auxiliary variable $z_{i}=x^{T}x-x_{i}^{2}$, and consider the function $y_{i}=f\left(x_{i},z_{i}\right)=x_{i}/\sqrt{x_{i}^{2}+z_{i}}$. The function $f\left(x_{i},z_{i}\right)$ has relatively simple second derivatives, and the variable $z_{i}$ has known moments, allowing us to use Taylor expansions to approximate the first moment $\gamma_{i}=E\left[f\left(x_{i},z_{i}\right)\right]$. Taking the expectation of the second-order Taylor expansion of $f\left(x_{i},z_{i}\right)$ around the point $\left[\mu_{i},{\bar{z}}_{i}\right]$ gives

(2) $$\gamma_{i}\;=E\left[f\left(x_{i},z_{i}\right)\right]\approx \frac{\mu_{i}}{\sqrt{\mu_{i}\;+\;{\bar{z}}_{i}}}\;\;+{\left.\frac{\Sigma_{ii}}{2}\cdot \frac{\partial^{2}f}{\partial x_{i}^{2}}\right|}_{\begin{array}{c} x_{i}=\mu_{i}\\ z_{i}={\bar{z}}_{i} \end{array}}+{\left.\frac{var\left(z_{i}\right)}{2}\cdot \frac{\partial^{2}f}{\partial z_{i}^{2}}\right|}_{\begin{array}{c} x_{i}=\mu_{i}\\ z_{i}={\bar{z}}_{i} \end{array}}\;\;+{\left.cov\left(x_{i},z_{i}\right)\cdot \frac{\partial^{2}f}{\partial x_{i}\partial z_{i}}\right|}_{\begin{array}{c} x_{i}=\mu_{i}\\ z_{i}={\bar{z}}_{i} \end{array}}$$

Solving for the second derivatives yields

(3) $$\gamma_{i}\approx \frac{\mu_{i}}{\sqrt{\mu_{i}^{2}\;+\;{\bar{z}}_{i}}}\;+\;\frac{\Sigma_{ii}}{2}\cdot \left(\frac{-3\mu_{i}{\bar{z}}_{i}}{{\left(\mu_{i}^{2}\;+\;{\bar{z}}_{i}\right)}^{5/2}}\right)\;\;+\frac{var\left(z_{i}\right)}{2}\cdot \left(\frac{3\mu_{i}}{4{\left(\mu_{i}^{2}+{\bar{z}}_{i}\right)}^{5/2}}\right)\;\;+cov\left(x_{i},z_{i}\right)\cdot \left(\frac{\mu_{i}^{2}\;-\;\frac{1}{2}{\bar{z}}_{i}}{{\left(\mu_{i}^{2}\;+\;{\bar{z}}_{i}\right)}^{5/2}}\right)$$

The variable $z_{i}$ is a quadratic form of a Gaussian random variable, and there are known formulas for $\bar{z_{i}}$, $var\left(z_{i}\right)$ and $cov\left(x_{i},z_{i}\right)$ (Mathai and Provost 1992) (see Appendix A). Denoting the vector μ without the i-th element as $\mu_{-i}$, and the covariance matrix Σ without the i-th row and column as $\Sigma_{-i}$, we have

$$\;{\bar{z}}_{i}\;=tr\left(\Sigma_{-i}\right)+\mu_{-i}^{T}\mu_{-i}\;\;var\left(z_{i}\right)\;=2tr\left(\Sigma_{-i}\Sigma_{-i}\right)+4\mu_{-i}^{T}\Sigma_{-i}\mu_{-i}\;cov\left(x_{i},z_{i}\right)\;=2\left(\mu^{T}{col}_{i}(\Sigma )-\mu_{i}\Sigma_{ii}\right)$$

where ${col}_{i}(\Sigma )$ is the i-th column of Σ.

With straightforward algebraic manipulations, a vectorized formula for the first moment can be obtained (see Appendix B).

#### 2.2.2 Second moment and covariance approximation

Next, we approximate the (non-centered) second moment matrix. To approximate the i, j element $E\left[y_{i}y_{j}\right]=E\left[x_{i}x_{j}/x^{T}x\right]$, consider the two variables $n_{ij}=x_{i}x_{j}$ and $d=x^{T}x$, and the function $y_{i}y_{j}=g\left(n_{ij},d\right)=n_{ij}/d$. The variables $n_{ij}$ and d are both quadratic forms of the Gaussian random variable x, and $g\left(n_{ij},d\right)$ is a ratio of quadratic forms. Computing the expected value of ratios of quadratic forms is a well-studied problem, and the following second-order Taylor approximation around the point $\left[{\overset{‾}{n}}_{ij},\overset{‾}{d}\right]$ is reported to have high accuracy (Paolella 2003).

(4) $$E\left[y_{i}y_{j}\right]=E\left[g\left(n_{ij},\;d\right)\right]\approx \;\;\frac{{\overset{‾}{n}}_{ij}}{\overset{‾}{d}}\left(1-\frac{cov\left(n_{ij},\;d\right)}{{\overset{‾}{n}}_{ij}\overset{‾}{d}}+\frac{var(d)}{{\overset{‾}{d}}^{2}}\right)$$

The moments for $n_{ij}$ and d can be computed using formulas for the moments of quadratic forms (Mathai and Provost 1992), resulting in

$$\;{\overset{‾}{n}}_{ij}=\Sigma_{ij}+\mu_{i}\mu_{j}\;\;\overset{‾}{d}=tr(\Sigma )+\mu^{T}\mu \;\;var\left(d\right)=2\;tr(\Sigma \Sigma )+4\mu^{T}\Sigma \mu \;cov\left(n_{ij},d\right)=2tr\left(\Sigma A^{ij}\Sigma \right)+4\mu^{T}A^{ij}\Sigma \mu$$

where $A^{ij}$ is an n-by-n matrix where all elements are 0 , except the elements ij and ji that are equal to 1/2 in the case i≠j, and 1 in the case i=j. Vectorized formulas can be obtained for computing the full second moment matrix (see Appendix B).

Finally, the covariance matrix $\tilde{\Psi }$ is obtained using the formula $\Psi =E\left[{yy}^{T}\right]-\gamma \gamma^{T}$, with the approximations for the first and second moments above.

#### 2.2.3 Exact moments for $𝒫𝒩\left(\mu ,\sigma^{2}I\right)$

The special case where $\Sigma =\sigma^{2}I$ is important in the literature (Pewsey and García-Portugués 2021). For this special case, we derived the following exact formulas for the mean and covariance of y

(5) γ=a⋅μ

(6) $$\Psi =b\cdot \mu \mu^{T}+c\cdot I$$

where

a=Γn2+122σ2Γn2+1F1112;n+22;-‖μ‖22σ2b=1σ2(n+2)F111;n+42;-‖μ‖22σ2-a2c=1nF111;n+22;-‖μ‖22σ2

and F11(a;b;z) is the confluent hypergeometric function. See derivation in the Appendix C.

## 3 Accuracy of moments approximation for 𝒫𝒩(μ,Σ)

### 3.1 Approximation accuracy for 𝒫𝒩(μ,Σ)

In this section, we test the accuracy of the approximated moments, denoted $\tilde{\gamma }$ and $\tilde{\Psi }$, against the true moments γ and Ψ of the distribution.

#### 3.1.1 Methods

For a given projected normal distribution 𝒫𝒩(μ,Σ), we obtained the true moments γ and Ψ by sampling 10^6^ points from the distribution and computing their mean and covariance. To evaluate the approximations obtained with the formulas, we compared their results to the true moments obtained by sampling. We evaluated the approximations for distributions with dimensionality n=3, n=6, n=12, n=24, and n=48, and different values of a variance scale parameter s that controls the overall variance (see below for details).

We evaluated the approximation accuracy in two ways. The first is the relative mean squared error across the elements of the distribution moments. This metric is obtained by computing the sum of the squared differences between the approximated and true moments, and dividing it by the sum of the squared elements of the true moments:

$${\text{Error}}_{\gamma }\%=100\cdot \frac{\|\tilde{\gamma }\;-\;\gamma {\|}^{2}}{\|\gamma {\|}^{2}}$$

$${\text{Error}}_{\Psi }\%=100\cdot \frac{\|\tilde{\Psi }\;-\;\Psi {\|}_{F}^{2}}{\|\Psi {\|}_{F}^{2}}$$

where $\|\cdot {\|}_{F}$ is the Frobenius norm.

The second metric is the cosine similarity between the approximated and the true moments

$${cosine}_{\gamma }=\frac{\tilde{\gamma }\;\cdot \;\gamma }{\left\|\tilde{\gamma }\|\|\gamma \right\|}$$

$${cosine}_{\Psi }=\frac{tr(\Psi \tilde{\Psi })}{\|\tilde{\Psi }{\|}_{F}\|\Psi {\|}_{F}}$$

The cosine similarity is a measure of the angle between two vectors. It is equal to 1.0 when the vectors are parallel, and 0.0 when they are orthogonal. A cosine similarity close to 1.0 indicates that the structure of the approximated and the true moments is similar, without taking into account their scale.

For each dimensionality n and variance scale s, we sampled 100 pairs of parameters μ and Σ. The parameter μ was uniformly sampled on the unit sphere. The parameter Σ was obtained by randomly sampling a diagonal matrix D with positive entries (the eigenvalues of Σ) and a random orthonormal matrix V (with the eigenvectors as columns), such that $\Sigma ={VDV}^{T}$.

The elements of D were first sampled independently from the exponential distribution^2^ Exp(1). Then a constant of 0.01 was added for stability, and D was scaled by a factor $s^{2}/n$. The scalar s controls the overall scale of the covariance matrix Σ. Division by n was used to keep a constant ratio between the average value of $D_{ii}$ and the average squared value $\mu^{2}$, the latter of which scales with 1/n because of the constraint ‖μ‖=1. We used values of s=0.125, s=0.25, s=0.5 and s=1.0.

To sample the eigenvectors in the columns of V, we first generated a skew symmetric matrix S, where each lower diagonal element was independently sampled from a normal distribution. Then V was obtained by taking the matrix exponential of S.

#### 3.1.2 Results

**Fig. 2:**
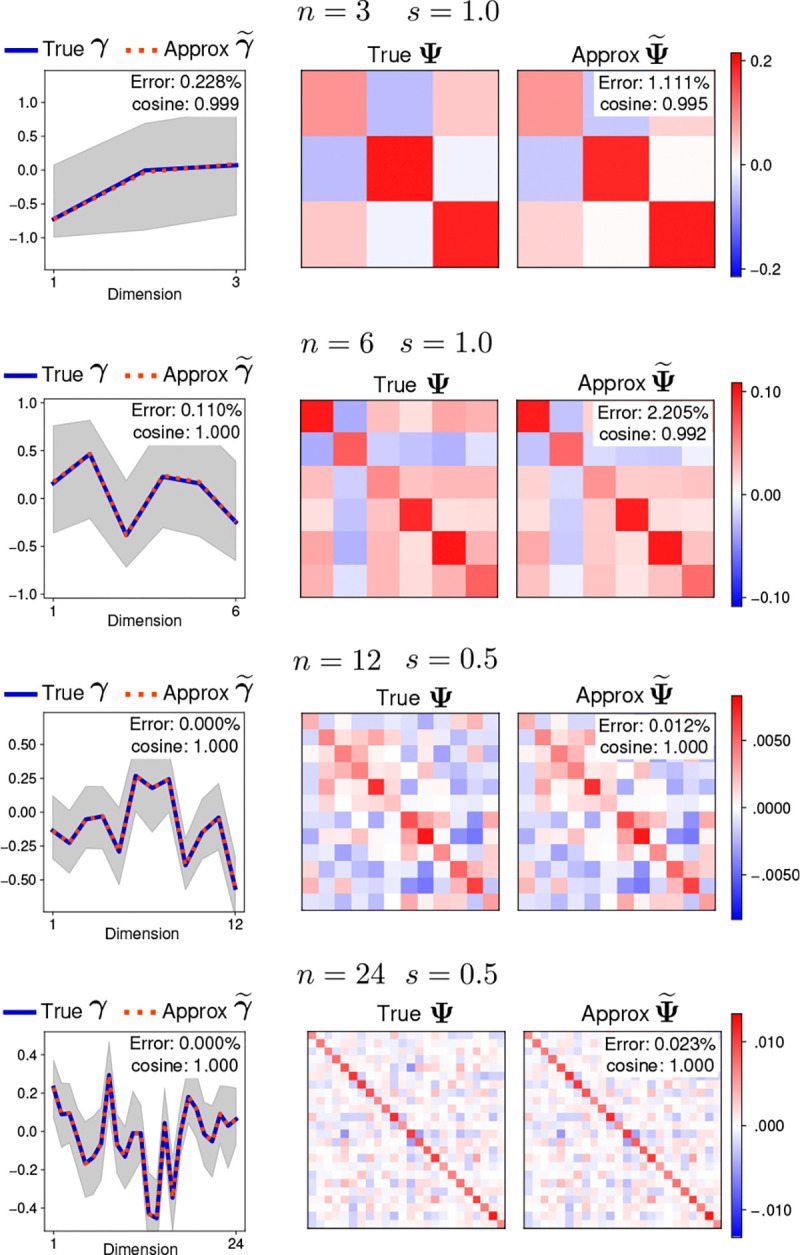
Moments approximation examples for 𝒫𝒩(μ,Σ). Each row shows the true and the approximated moments for a projected normal distribution of a different dimensionality n and scale s. The left column shows the true mean γ (blue) and the approximated mean $\tilde{\gamma }$ (red). The two means overlap almost perfectly. The shaded area shows the 95% quantile interval of 500 samples from the distribution. The right column shows the true covariance Ψ (left) and the approximated covariance $\tilde{\Psi }$ (right). Insets show the approximation error and cosine similarity for each example.

Figure 2 shows examples of the approximated and true moments γ and Ψ for different dimensionalities n and covariance scales s. The true and approximated moments are almost identical in all cases. The largest relative squared error is smaller than 1% for all the means and 3% for the covariances. The cosine similarities are all larger than 0.99. These results indicate that the approximated moments are very similar to the true moments. Visual inspection shows that the approximated parameters are almost identical to the true parameters, even for the largest errors.

**Fig. 3:**
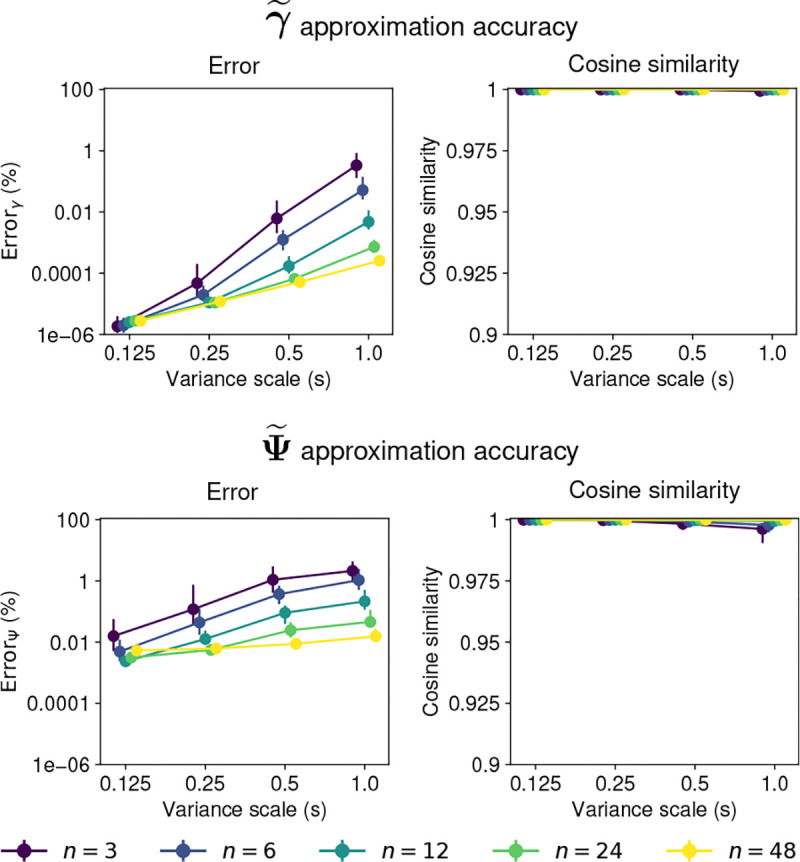
Accuracy of moments approximation for 𝒫𝒩(μ,Σ). First row shows the approximation error for the first moment $\tilde{\gamma }$. Second row shows the approximation error for the covariance $\tilde{\Psi }$. The left panel in each row shows the relative mean squared error with a log-scale y-axis, and the right panel shows the cosine similarity. Points show the median and lines show the interquartile range for 100 samples of the parameters. Colors indicate the dimension n of the distribution. The x-axis shows the scale factor s of the parameter Σ.

Figure 3 summarizes the approximation errors for different dimensionalities and covariance scales. Two features of the approximation error emerge from these plots. First, the approximation error for both $\tilde{\gamma }$ and $\tilde{\Psi }$ increases with the variance scale s. Second, the error tends to decrease with the dimensionality of the distribution.

The approximation error is small for both $\tilde{\gamma }$ and $\tilde{\Psi }$, particularly in the higher dimensional cases. For most dimensionalities and covariance scales, the median error for both $\tilde{\gamma }$ and $\tilde{\Psi }$ is smaller than 1%, and the median cosine similarity between the approximated and true moments is larger than 0.99.

Hence, the approximation method is accurate for obtaining the first and second moments of the projected normal distribution. The accuracy is particularly high for larger dimensions, and for cases where the covariance scale is not large.

### 3.2 Moment matching for 𝒫𝒩(μ,Σ)

In this section, we use the moment approximations to fit the parameters μ and Σ of the projected normal distribution 𝒫𝒩(μ,Σ) via moment matching.

#### 3.2.1 Methods

We generated 50 samples samples of μ and Σ using the same procedure as in Section 3.1.1. For a given pair of parameters μ and Σ, we sampled 10^7^ points from 𝒫𝒩(μ,Σ), and computed the moments γ and Ψ, which are denoted as the observed moments in the following. The goal of the moment matching procedure is to find the model parameters $\hat{\mu }$ and $\hat{\Sigma }$ such that the moments of the corresponding distribution, $\tilde{\gamma }$ and $\tilde{\Psi }$, obtained with our approximation formulas, are closest to the observed γ and Ψ.

To optimize the parameters $\hat{\mu }$ and $\hat{\Sigma }$, we use the mean squared difference between the observed and the approximaed moments as a loss function:

(7) $$\underset{\hat{\mu }\in S^{n-1},\hat{\Sigma }\in SPD}{argmin}(1-\lambda )\|\tilde{\gamma }-\gamma {\|}^{2}+\lambda \|\tilde{\Psi }-\Psi {\|}_{F}^{2}$$

where λ is hyperparameter that controls the relative weight of the mean and the covariance in the loss function (see below for details on the parameter constraints). Unless otherwise specified, we set λ=0.9.

To evaluate the fit quality, we used the same metrics as in Section 3.1.1. We used the relative mean squared error between the true and the fitted parameters:

$${\text{Error}}_{\mu }\%=100\frac{\|\hat{\mu }\;-\;\mu {\|}^{2}}{\|\mu {\|}^{2}}$$

$${\text{Error}}_{\Sigma }\%=100\frac{\|\hat{\Sigma }\;-\;\Sigma {\|}_{F}^{2}}{\|\Sigma {\|}_{F}^{2}}$$

and we also used the cosine similarity:

$${cosine}_{\mu }=\frac{\hat{\mu }\;\cdot \;\mu }{\left\|\hat{\mu }\|\|\mu \right\|}$$

$${cosine}_{\Sigma }=\frac{tr(\hat{\Sigma }\Sigma )}{\|\hat{\Sigma }{\|}_{F}\|\Sigma {\|}_{F}}$$

#### 3.2.2 Optimization

The NAdam optimizer in PyTorch (Paszke et al. 2017) was used to optimize Equation (7). A cyclic schedule was used for the learning rate. The first cycle had 80 iterations with an initial learning rate of 0.4, which was multiplied by a decay rate of 0.85 every 5 iterations. At the end of the 80 iterations, the next cycle started, with a learning rate of 0.85 times the starting learning rate of the previous cycle. Other than the starting learning rate, the new cycle is identical to the first. A full optimization schedule used 12 such cycles.

This optimization problem is not convex, so it is important to use a good initialization of the parameters. The model parameter $\hat{\mu }$ was initialized to γ/‖γ‖, and the parameter $\hat{\Sigma }$ was initialized to Ψ.

#### 3.2.3 Parameterization

The parameters μ and Σ of the projected normal distribution are not uniquely determined. Scaling x by a positive constant (with the corresponding changes in μ and Σ) does not change the distribution of y. Constraints are needed to make the parameters identifiable.

Previous work has placed the identifiability constraints on the covariance matrix $\hat{\Sigma }$ (Wang and Gelfand 2013; Hernandez-Stumpfhauser et al. 2017; Paine et al. 2018). Here, we constrain $\hat{\mu }$ to be on the unit sphere, i.e. ‖μ‖=1, and constrain $\hat{\Sigma }$ to be in the manifold of symmetric positive definite (SPD) matrices (see Equation 7). To constrain $\hat{\Sigma }$, we use the Python package geotorch for optimization on manifolds using trivializations (Lezcano-Casado and Martínez-Rubio 2019; Lezcano Casado 2019).

#### 3.2.4 Results

**Fig. 4:**
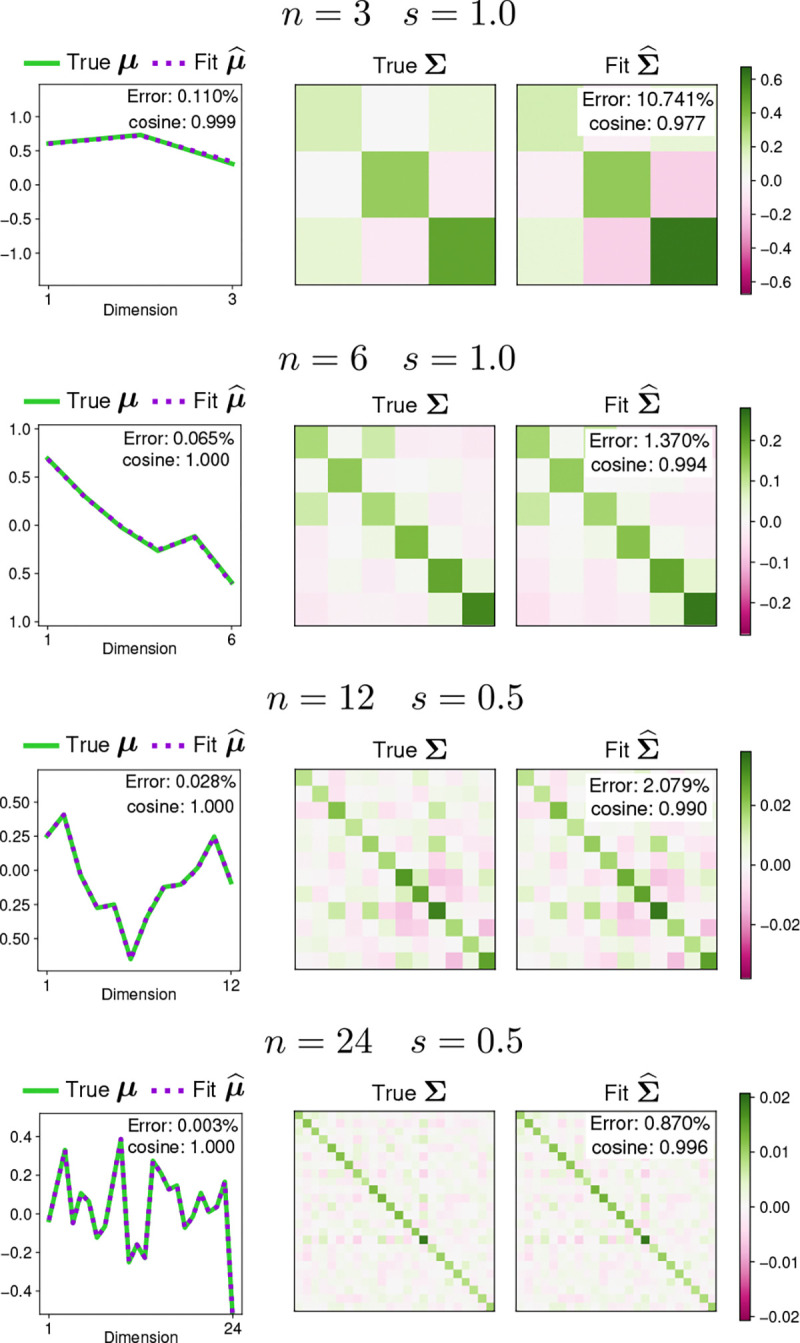
Moment matching examples for 𝒫𝒩(μ,Σ). Each row shows the true and the fitted parameters for a projected normal distribution of a different dimensionality n and scale s. The left column shows the true mean parameter μ (purple) and the fitted mean parameter $\hat{\mu }$ (green). The two means overlap almost perfectly. The right column shows the true covariance parameter Σ (left) and the fitted covariance parameter $\hat{\Sigma }$ (right) using a color scale. Insets show the fit error and cosine similarity for each example.

Figure 4 shows examples of the parameter fits for different dimensionalities and covariance scales. The fitted parameter $\hat{\mu }$ is almost identical to the true parameter μ in all examples, which is reflected in low errors and high cosine similarities. The fitted parameter $\hat{\Sigma }$ is very similar to the true Σ in all examples. The errors for $\hat{\Sigma }$ are larger, with an example showing a relative errors around 10%. The cosine similarities are close to 1.0 in all cases, in line with visual inspection showing that $\hat{\Sigma }$ largely captures the structure of the true Σ.

**Fig. 5:**
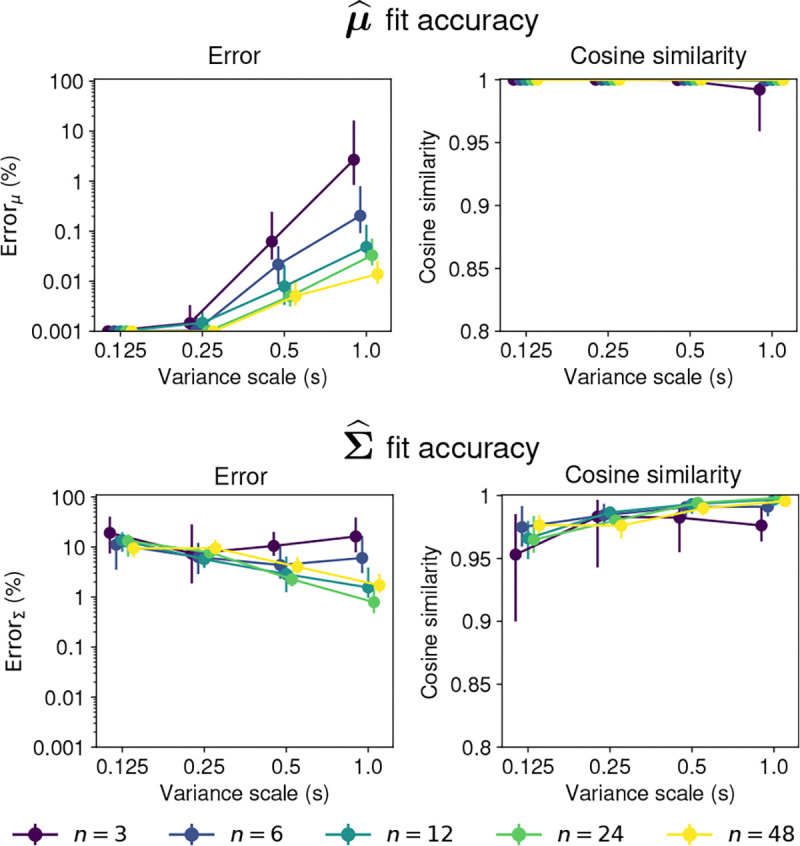
Accuracy of moment matching for 𝒫𝒩(μ,Σ). First row shows the fit error for the mean parameter $\hat{\mu }$. Second row shows the fit error for the covariance parameter $\hat{\Sigma }$. The left panel in each row shows the relative mean squared error with a log-scale *y*-axis, and the right panel shows the cosine similarity Points show the median and lines show the interquartile range for 50 samples of the parameters. Colors indicate the dimension n of the distribution. The x-axis shows the scale factor s of the parameter Σ.

The median error and cosine similarity for the moment matching fit are shown in Figure 5. Similar to the moment approximation (Figure 3), the quality of the fit tends to increase with the dimensionality n. For the mean parameter $\hat{\mu }$, the error also increases with the covariance scaling factor s. Conversely, the error for $\hat{\Sigma }$ decreases as s increases. The error in the parameter fits is in general smaller than 10% for most values of n and s, and in many cases are in the order of 1%. The cosine similarity for $\hat{\mu }$ is close to 1.0 for all values of n and s. For $\hat{\Sigma }$, the median cosine similarity is larger than 0.95 for all values of n and s. Overall, the moment matching procedure is able to estimate the model parameters with an accuracy that depends on the dimensionality n and the covariance scale s, and that is high for most conditions.

## 4 Generalizations of the projected normal distribution

In this section, we present three generalizations of the projected normal distribution. Each of these generalizations uses a different denominator for divisive normalization, and has a different geometrical interpretation (Figure 1). For each of the generalizations, we derive the moments approximation, and when possible, the probability density function.

### 4.1 Denominator with an additive constant: Projecting inside the unit sphere

The first extension that we consider is the random variable $y=x/\sqrt{x^{T}x+c}$, which includes a positive constant c in the denominator. Such additive constants are a common feature of divisive normalization models in neuroscience (Albrecht and Geisler 1991; Schwartz and Simoncelli 2001; Carandini and Heeger 2012). We denote the distribution of this random variable as $𝒫{𝒩}_{c}(\mu ,\Sigma ,c)$.

One key difference between 𝒫𝒩(μ,Σ) and $𝒫{𝒩}_{c}(\mu ,\Sigma ,c)$ is that the former is defined on the surface of the unit sphere (i.e. for y such that ‖y‖=1), whereas the latter is defined in its interior (i.e. for y such that ‖y‖<1) (Figure 1).

#### 4.1.1 Probability density function

Although the focus of this work is on the moments of the projected normal distribution and its extensions, we provide formulas for the probability density function of the novel distributions for completeness. Here, we derive the probability density function of $𝒫{𝒩}_{c}(\mu ,\Sigma ,c)$.

Let $h(x)=x/\sqrt{x^{T}x+c}$ denote the invertible transformation that maps x into y. The variables x and y have the same direction but different norms, because y is obtained by dividing x by a positive scalar. Thus, the inverse of h is given by $h^{-1}(y)=y(\|x\|/\|y\|)=x$. To compute $h^{-1}(y)$, we need to solve for ‖x‖ in terms of y.

Noting that $\|y\|=\|x\|/\sqrt{\|x{\|}^{2}+c}$, solving for ‖x‖ results in

$$\|x\|=\|y\|\sqrt{\frac{c}{1\;-\;\|y{\|}^{2}}}$$

Thus, we can write the inverse transformation as

(8) $$x=h^{-1}(y)=y\sqrt{\frac{c}{1\;-\;\|y{\|}^{2}}}$$

The Jacobian matrix for $h^{-1}(y)$ can be easily computed by applying the chain rule, resulting in

$$J=\sqrt{\frac{c}{1\;-\;\|y{\|}^{2}}}\left(I+\frac{{yy}^{T}}{1\;-\;\|y{\|}^{2}}\right)$$

A simple formula for det(J) can be obtained by using the properties $det\left(kA\right)=k^{n}det(A)$ for a scalar k, and $det\left(I+{vv}^{T}\right)=1+\|v{\|}^{2}$, resulting in

(9) $$det(J)={\left(\frac{c}{1\;-\;\|y{\|}^{2}}\right)}^{n/2}\left(1+\frac{\left\|y^{2}\right\|}{1\;-\;\|y{\|}^{2}}\right)$$

Finally, using the change of variable formula, we obtain

(10) $$p(y;\mu ,\Sigma ,c)=\frac{det(J)}{det(2\pi \Sigma {)}^{\frac{1}{2}}}\;\;\times e^{-\frac{1}{2}{\left(h^{-1}(y)-\mu \right)}^{T}\Sigma^{-1}\left(h^{-1}(y)-\mu \right)}$$

Like for 𝒫𝒩(μ,Σ), the formulas for the density function can be used to fit the distribution parameters using maximum likelihood estimation through iterative methods. We leave this task for future work, and focus on the moments of the distributions and moment matching procedures.

#### 4.1.2 Approximation to the moments of $𝒫{𝒩}_{c}(\mu ,\Sigma ,c)$

To approximate the first and second moments of $y\sim 𝒫{𝒩}_{c}(\mu ,\Sigma ,c)$ we use the same Taylor expansion approach that we describe in Section 2.2. The same formulas require minor modifications to account for the constant c in the denominator.

To approximate $\gamma_{i}$, we consider the same function as in Section 2.2.1, $y_{i}=f\left(x_{i},z_{i}\right)=x_{i}/\sqrt{x_{i}^{2}+z_{i}}$ where $z_{i}=x^{T}x-x_{i}^{2}+c$. The second derivatives of f and thus Equation (3) are unchanged. The expression forthe expected value of $z_{i}$ is the only change, which is now given by

(11) $${\bar{z}}_{i}=tr\left(\Sigma^{i}\right)+\mu^{iT}\mu^{i}+c$$

Therefore, to approximate $\gamma_{i}$, we use all the same formulas as in Section 2.2.1, except that we use Equation 11 to obtain $\bar{z}$.

Similarly, to approximate the second moment matrix we consider the same function as in Section 2.2.2, $y_{i}y_{j}=g\left(n_{ij},d\right)=n_{ij}/d$, where $d=x^{T}x+c$ and $n_{ij}=x_{i}x_{j}$. Again, the second derivatives of g and thus Equation (4) are unchanged. Only the expected value of d changes, which is now given by

(12) $$\overset{‾}{d}=tr(\Sigma )+\mu^{T}\mu +c$$

Thus, the second moment can be approximated using the same formulas as in Section 2.2.2, except that we use Equation 12 to obtain $\overset{‾}{d}$.

### 4.2 Denominator with $x^{T}Bx$ quadratic form: Projecting to an ellipsoid surface

Next, we consider the random variable $y=x/\sqrt{x^{T}Bx}$ where B is a SPD matrix and $x^{T}Bx$ is a quadratic form of x. In neuroscience, models with such denominators have been successful in describing complex interactions between neurons (Coen-Cagli and Schwartz 2013; Coen-Cagli et al. 2012). We denote this distribution as $𝒫{𝒩}_{B}(\mu ,\Sigma ,B)$.

Geometrically, the variable y defined above satisfies $y^{T}By=1$. Thus y is constrained to be on the surface of an ellipsoid defined by B (Figure 1).

Importantly, the random variable $y\sim 𝒫{𝒩}_{B}(\mu ,\Sigma ,B)$ can be obtained by linear transformation of a variable $y^{\prime }\sim 𝒫𝒩\left(\mu^{\prime },\Sigma^{\prime }\right)$. Let $x^{\prime }=B^{1/2}x$, where $B^{1/2}$ is the matrix square root of B^3^. Then we have

(13) $$y\;=\frac{x}{\sqrt{x^{T}Bx}}\;\;=B^{-1/2}\frac{x^{\prime }}{\sqrt{x^{\prime T}x^{\prime }}}\;\;=B^{-1/2}y^{\prime }$$

where $y^{\prime }=x^{\prime }/\sqrt{x^{\prime T}x^{\prime }}$. The variable $x^{\prime }$ follows a normal distribution $x^{\prime }\sim 𝒩\left(\mu^{\prime },\Sigma^{\prime }\right)$, where $\mu^{\prime }=B^{1/2}\mu$ and $\Sigma^{\prime }=B^{1/2}\Sigma B^{1/2}$. Therefore, the variable $y^{\prime }$–which is is linearly related to y–follows a projected normal distribution. This fact can be used to obtain the moments of y.

#### 4.2.1 Probability density function of $𝒫{𝒩}_{B}(\mu ,\Sigma ,B)$

The simple linear relation between the variable $y\sim 𝒫{𝒩}_{B}(\mu ,\Sigma ,B)$ and the variable $y^{\prime }\sim 𝒫𝒩\left(\mu^{\prime },\Sigma^{\prime }\right)$ with a known density function (Equation 1) might suggest to the reader that the density function of y can be easily obtained with the change of variable formula. However, this is not the case. Because y and $y^{\prime }$ are defined on n-1 dimensional surfaces, applying the change of variable formula requires computing how the linear transformation $B^{-1/2}$ changes the local area element at any arbitrary point of the sphere on which $y^{\prime }$ is defined. This is a non-trivial task that we leave for future work.

#### 4.2.2 Approximation to the moments of $𝒫{𝒩}_{B}(\mu ,\Sigma ,B)$

To approximate the moments of y we use the relation $y=B^{-1/2}y^{\prime }$, where $y^{\prime }$ follows a projected normal distribution. First, the parameters $\mu^{\prime }$ and $\Sigma^{\prime }$ are computed as mentioned in Section 4.2. Then, the first and second moments of $y^{\prime }$, $\gamma^{\prime }$ and $\Psi^{\prime }$ respectively, are computed using the formulas derived in Section 2.2. Finally, the moment approximations of y are computed using the formulas $\gamma =B^{-1/2}\gamma^{\prime }$ and $\Psi =B^{-1/2}\Psi^{\prime }B^{-1/2}$.

### 4.3 Denominator with a quadratic form and an additive constant: Projecting inside an ellipsoid

Finally, we consider the random variable $y=x/\sqrt{x^{T}Bx+c}$, where B is a SPD matrix and c is a positive constant. We denote the distribution of this random variable as $𝒫{𝒩}_{Bc}(\mu ,\Sigma ,B,c)$. Geometrically, the projected variable y defined above satisfies $y^{T}By<1$, which means that y is in the interior of an ellipsoid defined by B (Figure 1).

The variable y is related to a variable $y^{\prime }\;\sim \;𝒫{𝒩}_{c}\left(\mu^{\prime },\Sigma^{\prime },c\right)$ (Section 4.1) via a linear transformation. Defining $x^{\prime }=B^{1/2}x$, we have

(14) $$y\;=\frac{x}{\sqrt{x^{T}Bx\;+\;c}}\;\;=B^{-1/2}\frac{x^{\prime }}{\sqrt{x^{\prime T}x^{\prime }\;+\;c}}\;\;=B^{-1/2}y^{\prime }$$

where $y^{\prime }=x^{\prime }/\sqrt{x^{\prime T}x^{\prime }+c}$. The variable $x^{\prime }$ has a distribution $x^{\prime }\sim 𝒩\left(\mu^{\prime },\Sigma^{\prime }\right)$, with $\mu^{\prime }=B^{1/2}\mu$ and $\Sigma^{\prime }=B^{1/2}\Sigma B^{1/2}$, implying that $y^{\prime }\;\sim \;𝒫{𝒩}_{c}\left(\mu^{\prime },\Sigma^{\prime },c\right)$.

#### 4.3.1 Probability density function

The density function of $y\sim 𝒫{𝒩}_{Bc}(\mu ,\Sigma ,B,c)$ can be obtained from the density function of $y^{\prime }\sim 𝒫{𝒩}_{c}\left(\mu^{\prime },\Sigma^{\prime },c\right)$ presented in Equation 10. Using the change of variable formula for linear transformations, we have

(15) $$p(y;\mu ,\Sigma ,B,c)=\;\;p(B^{-1/2}y;\mu^{\prime },\Sigma^{\prime },c)det(B^{1/2})$$

#### 4.3.2 Approximation to the moments of $𝒫{𝒩}_{Bc}(\mu ,\Sigma ,\;B,c)$

To approximate the moments of $y\;\sim \;𝒫{𝒩}_{Bc}(\mu ,\Sigma ,B,c)$ we use the fact that y is a linear transformation of $y^{\prime }\;\sim \;𝒫{𝒩}_{c}\left(\mu^{\prime },\Sigma^{\prime },c\right)$ (see Section 4.2.2). The moments can be computed using the formulas in Section 4.1.2. First, we compute the parameters $\mu^{\prime }$ and $\Sigma^{\prime }$ as described above. Then, we compute the first and second moments of $y^{\prime }$, $\gamma^{\prime }$ and $\Psi^{\prime }$, using the formulas in Section 4.1.2. Finally, we obtain the moments of y using the transformations $\gamma =B^{-1/2}\gamma^{\prime }$ and $\Psi =B^{-1/2}\Psi^{\prime }B^{-1/2}$.

## 5 Testing the moments approximation for generalizations of the projected normal

In this section, we test the accuracy of the moments approximation and moment matching for the distribution $𝒫{𝒩}_{Bc}(\mu ,\Sigma ,B,c)$. The approximation accuracy and the moment matching accuracy for the distributions $𝒫{𝒩}_{c}(\mu ,\Sigma ,c)$ and $𝒫{𝒩}_{B}(\mu ,\Sigma ,B)$ are available in the Supplementary materials. In all cases, the conclusions are the same: the Taylor-expansion-based approximations for moments are accurate.

### 5.1 Approximation accuracy for $𝒫{𝒩}_{Bc}(\mu ,\Sigma ,\;B,c)$

#### 5.1.1 Methods

We generated 100 random samples of the parameters μ, Σ, B and c to test the accuracy of the moment approximations. The parameters μ and Σ were sampled in the same way as in Section 3.1.1.

Importantly, the effect of the parameter c depends on its scale relative to $\|x{\|}^{2}$. At the same time, $E(\|x{\|}^{2})$ depends on the parameters n, μ, and Σ. To make the effect of c comparable across conditions, we sampled a multiplier value $c_{\mathrm{mult}}$ from the exponential distribution Exp(1), and then we defined the constant in the denominator as $c=c_{\mathrm{mult}}E(\|x{\|}^{2})$.

Samples of B were generated with the same procedure that was used to generate samples of Σ (i.e. we sampled the eigenvalues independently from Exp(1) and the eigenvectors as described in Section 3.1.1). Unlike Σ, the matrix B was not scaled by a scaling factor (i.e. s).

We used the same methods as in Section 3.1.1 to evaluate the accuracy of the moments approximation.

#### 5.1.2 Results

**Fig. 6:**
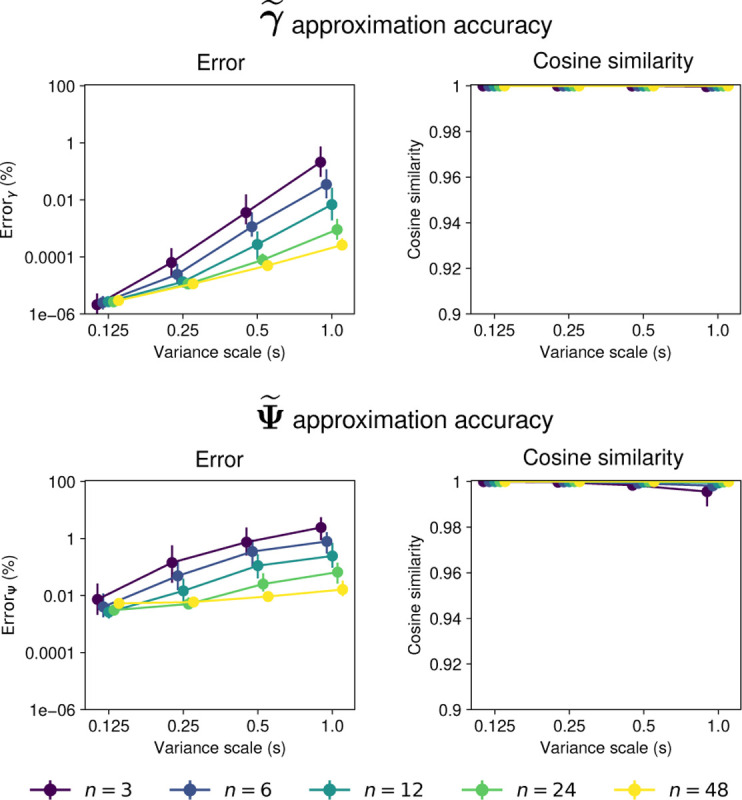
Accuracy of moments approximation for $𝒫{𝒩}_{Bc}(\mu ,\Sigma ,B,c)$. First row shows the approximation error for the first moment γ. Second row shows the approximation error for the covariance Ψ. The left panel in each row shows the relative mean squared error with a log-scale y-axis, and the right panel shows the cosine similarity. Points show the median and lines show the interquartile range for 100 samples of the parameters. Points are colored by the dimension n of the distribution. The x-axis shows the scale factor s of the parameter Σ.

Figure 6 shows the approximation accuracy for the moments of $𝒫{𝒩}_{Bc}(\mu ,\Sigma ,B,c)$. The approximations have high accuracy, with median relative errors for γ and Ψ smaller than 1% for most values of n and s, and median cosine similarities larger than 0.99. Like for the projected normal distribution, error decreases with n and increases with the scale s of the covariance (compare to Figure 3).

### 5.2 Moment matching for $𝒫{𝒩}_{Bc}(\mu ,\Sigma ,B,c)$

#### 5.2.1 Methods

In preliminary analyses, we observed that fitting the full parameter set μ, Σ, B, and c of the distribution $𝒫{𝒩}_{Bc}(\mu ,\Sigma ,B,c)$ via moment matching is difficult. This is likely due to the large number of parameters to be estimated, and the non-linear constraints imposed on these parameters. Therefore, we simplified the moment matching problem by adding further constraints to the parameters in both the sampling and optimization steps.

First, since the focus of this section is on fitting the denominator matrix B, to reduce the total number of free parameters we constrained the fitted model parameter $\hat{\Sigma }$ to be of the form $\hat{\Sigma }={\hat{\sigma }}^{2}I$, where ${\hat{\sigma }}^{2}$ is a free parameter and I is the identity matrix. The true Σ was sampled by first sampling the parameter $\sigma^{2}$ from the uniform distribution 𝒰(0.05,1.0), and then scaling it by the factor $s^{2}/n$. Then the true covariance matrix was obtained as $\Sigma =\sigma^{2}I$. Note that because of this constraint on Σ, the cosine similarity between the true and fitted covariance matrices, ${cosine}_{\Sigma }$, is always 1.0.

Second, we constrained the fitted model parameter $\hat{B}$ to be of the form $\hat{B}=I+\hat{b}\hat{v}\hat{v}$, where $\hat{b}$ and $\hat{v}$ are free parameters. The parameter $\hat{b}$ is constrained to be a positive number and $\hat{v}$ is constrained to have unit norm. The true B was obtained by sampling b as b=2+Exp(4) and v was sampled uniformly from the unit sphere, and then B was defined as $B=I+bvv^{T}$.

Although the constraints imposed on $\hat{\Sigma }$ and $\hat{B}$ in this section are arbitrary, for real-world problems knowledge about B and Σ can be leveraged to impose useful constraints that aid the optimization.

#### 5.2.2 Optimization

Preliminary analyses showed that fit accuracy depends on the covariance weighting hyperparameter λ (see Equation 7) in way that depends on the dimensionality n and the scale s of the covariance matrix. For example, in comparing hyperparameters λ=0.9 and λ=0.98, the former performed much better for n=3 and s=1.0, while the latter performed much better for n=48 and s=0.125. Thus, for each combination of n and s, we used the value of λ that leads to the smallest median relative squared error for the parameter B. We tested values of λ=0.66, λ=0.9, λ=0.95 and λ=0.98.

The model parameter ${\hat{\sigma }}^{2}$ was initialized to the value of tr(Ψ)/n, where Ψ is the observed covariance matrix of the data y. We initialized $\hat{v}$ to the eigenvector with the smallest eigenvalue of the observed Ψ, because the eigenvector v of B has the largest eigenvalue, and thus the the denominator therm $x^{T}Bx$ will attenuate the values of y in the direction of v. $\hat{\mu }$ was initialized to ${\hat{B}}^{1/2}\gamma /\|\gamma \|$.

#### 5.2.3 Results: $𝒫{𝒩}_{Bc}(\mu ,\Sigma ,B,c)$ moment matching

**Fig. 7:**
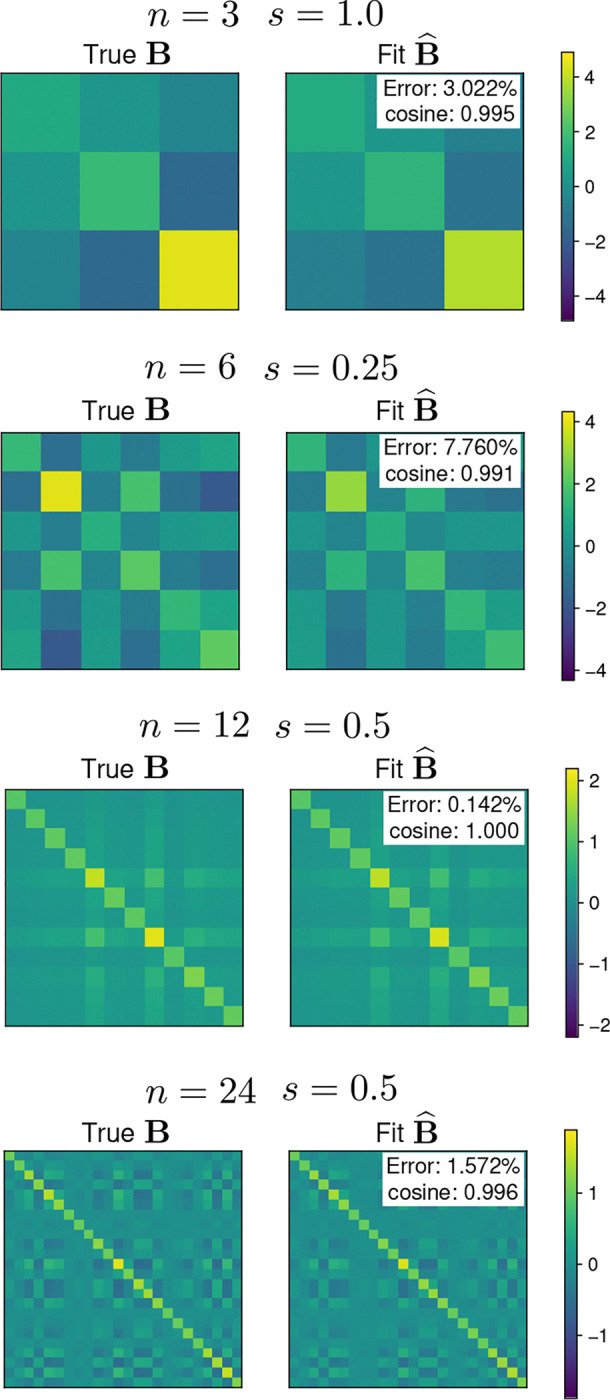
Moment matching examples for the denominator matrix B in $𝒫{𝒩}_{B}(\mu ,\Sigma ,B)$. Each panel shows the true parameter B (left) and the one obtained through moment matching $\hat{B}$ (right) for different values of n and s (indicated above each panel). Insets show the error and the cosine similarity for the fitted matrices $\hat{B}$.

Figure 7 shows examples of the true and the fitted B matrices for different dimensionalities and covariance scales. Across all examples, the cosine similarity between the true and fitted is close to 1.0, and the relative errors are smaller than 10%, indicating that the fitted $\hat{B}$ captures the structure of the true B well.

**Fig. 8:**
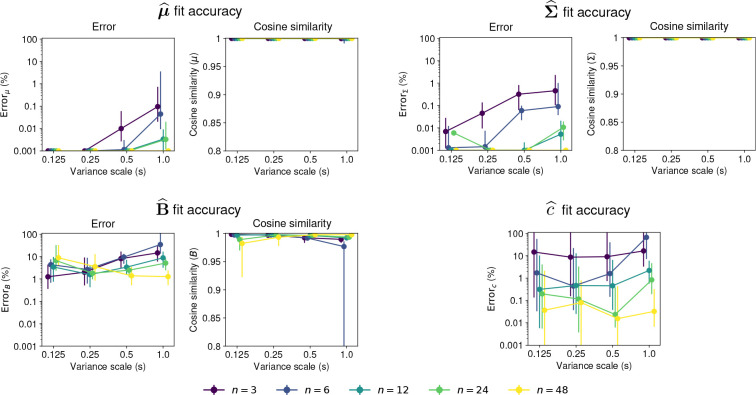
Accuracy of moments matching for $𝒫{𝒩}_{Bc}(\mu ,\Sigma ,B,c)$. First row shows the fit error for the mean parameter $\hat{\mu }$ (left panel) and for the covariance parameter $\hat{\Sigma }$ (right panel). Second row shows the fit error for the denominator matrix $\hat{B}$ (left panel) and for the denominator constant $\hat{c}$ (right panel). The left plot in each panel shows the relative mean squared error with a log-scale y-axis, and the right panel shows the cosine similarity Points show the median and lines show the interquartile range for 50 samples of the parameters. Colors indicate the dimension n of the distribution. The x-axis shows the scale factor s of the parameter Σ.

Figure 8 shows the moment matching fit accuracy. The accuracy for the fit is high for for both the mean $\hat{\mu }$ and the covariance $\hat{\Sigma }$, with median relative errors smaller than 1% for all values of n and s. The error for the denominator matrix $\hat{B}$ is also relatively small, with median errors smaller than 10% for most values of n and s, and median cosine similarities larger than 0.97 for all values of n and s. Interestingly, the effect of the scale s is dependent on n, with the error increasing with s for distributions with n=3, and decreasing with s for n=48. Finally, the errors for the denominator constant $\hat{c}$ showed a marked decrease with n, with median errors in the order of 10% for n=3, and smaller than 1% for most other conditions.

In sum, moment matching is able to accurately fit the parameters of the distribution $𝒫{𝒩}_{Bc}(\mu ,\Sigma ,B,c)$, including the denominator matrix B and the denominator constant c for most values of n and s, under the parameter constraints used in this section.

## 6 Discussion

The projected normal distribution is a flexible probability distribution on the unit sphere ${𝒮}^{n-1}$, that has seen a recent surge of interest in statistics. In this work, we present closed-form analytic approximations of the first and second moments of the projected normal distribution of arbitrary dimensionality. These approximations have high accuracy and are computationally efficient.

Furthermore, we show that the approximations can be used to fit the parameters of the distribution through a moment matching procedure, resulting in accurate parameter estimates across a wide range of values of dimensionality n, and of scale s of the covariance matrix Σ. The moment approximation and moment matching procedures are a useful addition to the set of statistical tools recently developed for the projected normal distribution. The simplicity of the moment matching procedure allows for fast and efficient fitting of the parameters that should make the projected normal distribution more accessible in practice. We make these approximations and the moment matching procedures available as a Python package named ‘projnormal’.

We also introduced three different generalizations of the projected normal distribution motivated by an influential model of neural activity from computational neuroscience, the divisive normalization model (Carandini and Heeger 2012; Goris et al. 2024; Herrera-Esposito and Burge 2024). Geometrically, while the projected normal distribution projects the random variable onto the unit sphere, these generalizations of the projected normal project the random variable to the surface or to the interior of an ellipsoid. For some of these generalizations, we derive the probability density function of the distribution. For all of the generalizations, we find moment approximations, and show that moment matching can be used to fit the parameters of the distributions.

Divisive normalization models have been successful in describing and predicting neural responses in a wide range of systems and experimental setups in neuroscience (Schwartz and Simoncelli 2001; Coen-Cagli et al. 2012; Carandini and Heeger 2012; Goris et al. 2024). The projected normal distribution and its generalizations presented here should provide a natural framework to extend divisive normalization models to account for the joint of effects of noisy inputs and normalization on the statistical properties of neural response. The moments of the projected normal distribution and its generalizations are particularly relevant given recent progress in estimating the moments of neural population responses across unobserved experimental conditions (Nejatbakhsh et al. 2023; Ding et al. 2023), producing rich sets of moments that can be used to fit the parameters of the distributions, for which the underlying data is not available.

Some challenges remain in the application of the projected normal distribution and its generalizations to real-world problems. For the generalizations using a quadratic form $x^{T}Bx$ in the denominator, the matrix B adds a large number of parameters to the distribution, rendering optimization difficult. In this work, we used a constraint on B to reduce the number of parameters and to improve the optimization, and also constrained the covariance matrix Σ to be isotropic. Future work should explore ways to improve the optimization of the parameters B and Σ. Other methods for parameterization, initialization, and/or optimization should be helpful towards these efforts. For concrete applications, knowledge about the problem can be used to add problem-specific constraints that can aid the optimization while still allowing for a flexible representation of the data.

## Footnotes

1 In the literature, the term projected normal sometimes refers to the special case Σ=I, where I is the identity matrix. Here we use the term projected normal distribution to refer to the distribution with arbitrary Σ.

2 An alternative sampling method where the eigenvalues were sampled from the uniform distribution U(0.05,1.0) instead of the exponential distribution was also tested, leading to similar results.

3 The same approach can be used for other SPD matrix decompositions, such as the Cholesky decomposition where $B=Ł{Ł}^{T}$ for a lower triangular matrix Ł with positive diagonal elements.

## Appendix A Moments of quadratic forms of Gaussian random variables

For convenience, we present here some useful formulas for the moments of quadratic forms of Gaussian random variables, that can be found in (Mathai and Provost 1992) Chapter 3.2.

Let $x\in R^{n}$ be a Gaussian random variable x~𝒩(μ,Σ), where $\mu \in R^{n}$ is the mean vector and $\Sigma \in R^{n\times n}$ is the covariance matrix. Let $M\in R^{n\times n}$ and $KR^{n\times n}$ be symmetric positive definite (SPD) matrices, and $b\in R^{n}$ be a vector. Then, $q=x^{T}Mx$ and $p=x^{T}Kx$ are two quadratic forms of x, and $l=b^{T}x$ is a linear function of x. The following formulas are useful for computing the moments of the projected normal distribution

(A1)
$$\overset{‾}{q}=tr(M\Sigma )+\mu^{T}M\mu$$

(A2)
$$var(q)=2tr(M\Sigma M\Sigma )+4\mu^{T}M\Sigma M\mu$$

(A3)
$$cov(q,p)=2tr(M\Sigma K\Sigma )+4\mu^{T}M\Sigma K\mu$$

(A4)
$$cov(q,l)=2\mu^{T}M\Sigma b$$

For the approximation of $\gamma_{i}$, we used the variables $x_{i}$ and $z_{i}=x^{T}x-x_{i}^{2}$. We can consider $x_{i}$ as a linear function of x given by $x_{i}=e_{i}^{T}x$, where $e_{i}$ is the canonical i vector (i.e. a vector with 1 in position i and 0 elsewhere). Note that $z_{i}$ is the sum of the squares of all the elements of x except for the i ťh element. We can define z as the quadratic form $z=x^{T}I_{-i}x$, where $I_{-i}$ is the same as the identity matrix everywhere, except in the i’th diagonal element where it is 0. Then, we can use the formulas above to obtain the moments required to approximate $\gamma_{i}$.

Using Equation A1 with $M=I_{-i}$, we get the formula for ${\bar{z}}_{i}$ in the main text. Using Equation A2 with $M=I_{-i}$, we get the formula for $var\left({\bar{z}}_{i}\right)$ in the main text. Finally, using Equation A4 with $M=I_{-i}$ and $b=e_{i}$, we get the formula for $cov\left(x_{i},z_{i}\right)$ in the main text.

Similarly, for the approximation of the second moment matrix element $E\left[y_{i}y_{j}\right]$, we used the variables $n_{ij}=x_{i}x_{j}$ and $d=x^{T}Ix$. We can consider $n_{ij}$ as a quadratic form of x given by $n_{ij}=x^{T}A^{ij}x$, where $A^{ij}$ is a matrix with entries i, j and j, i equal to 1/2 in the case i≠j, and 1 in the case i=j, and 0 elsewhere.

Using Equation A1 with $M=A^{ij}$, we obtain the formula for ${\overset{‾}{n}}_{ij}$ in the main text. Using Equation A1 with M=I we obtain the formula for $\overset{‾}{d}$ in the main text. Using Equation A2 with M=I, we obtain the formula for var(d) in the main text. Finally, using Equation A3 with $M=A^{ij}$ and K=I, we obtain the formula for $cov\left(n_{ij},d\right)$ in the main text.

## Appendix B Vectorized formulas for moments approximation

### B.1 Vectorized formulas for γ

In Section 2.2.1 of the main text, we provide formulas to approximate the i’th element of the first moment $\gamma_{i}$ of the projected normal distribution. To compute the approximation to the full vector γ, the approximation for each element i can be computed separately. However, the different elements $\gamma_{i}$ share many of the same computations, and thus it is possible to compute γ in an efficient vectorized way.

First, we define the following vectors containing the required moments for every $z_{i}$:

$$\;\bar{z}=\left[{\bar{z}}_{1},\ldots ,{\bar{z}}_{n}\right]\;\;var(z)=\left[var\left(z_{1}\right),\ldots ,var\left(z_{n}\right)\right]\;cov(x,z)=\left[cov\left(x_{1},z_{1}\right),\ldots ,cov\left(x_{n},z_{n}\right)\right]$$

A vectorized formula for γ can be obtained as follows (compare to Equation 3 in the main text):

$$\gamma \approx \;\frac{\mu }{{\left(\mu^{2}\;+\;\bar{z}\right)}^{\frac{1}{2}}}\;+\;\frac{diag(\Sigma )}{2}⊙\left(\frac{-3\mu ⊙\bar{z}}{{\left(\mu^{2}\;+\;\bar{z}\right)}^{5/2}}\right)\;\;+\frac{var(z)}{2}⊙\left(\frac{3\mu }{4{\left(\mu^{2}\;+\;\bar{z}\right)}^{5/2}}\right)\;\;+cov(x,z)⊙\left(\frac{\mu^{2}\;-\;\frac{1}{2}\bar{z}}{{\left(\mu^{2}\;+\;\bar{z}\right)}^{5/2}}\right)$$

where diag(Σ) is a vector with the diagonal elements of Σ, ⊙ is the Hadamard product and, abusing the notation, the powers and divisions involving vectors are element-wise.

Then, efficient vectorized formulas can be obtained for the vectors $\bar{z}$, var(z), and cov(x,z). For instance, consider the formula for ${\bar{z}}_{i}$ in the main text,

$${\bar{z}}_{i}=tr\left(\Sigma^{i}\right)+\mu^{iT}\mu^{i}$$

where $\Sigma^{i}$ is the covariance matrix Σ with the i’th diagonal element set to 0 , and $\mu^{i}$ is the mean vector μ with the i’th element set to 0. This is equivalent to the formula where we use the full covariance matrix Σ and the full mean vector μ, and then subtract the terms corresponding to the i’th element:

$${\bar{z}}_{i}=tr(\Sigma )+\mu^{T}\mu -\Sigma_{ii}-\mu_{i}^{2}$$

Thus, to compute each ${\bar{z}}_{i}$, we can compute the shared terms $tr(\Sigma )+\mu^{T}\mu$, and then subtract the i-specific terms $\Sigma_{ii}+\mu_{i}^{2}$. This leads to the vectorized formula

$$\bar{z}=1\left(tr(\Sigma )+\mu^{T}\mu \right)-\left(diag(\Sigma )+\mu^{2}\right)$$

where 1 is a vector of ones of length n, and the squaring of μ is element-wise.

Applying this approach to the formulas for $var\left(z_{i}\right)$ and $cov\left(x_{i},z_{i}\right)$ in the main text and using some algebraic manipulations, the following vectorized formulas can be obtained:

$$var(\bar{z})=1\left(2tr(\Sigma \Sigma )+4\mu^{T}\Sigma \mu \right)\;\;-2\left(2diag(\Sigma \Sigma )-diag(\Sigma {)}^{2}\right)\;\;-4\left(2\mu ⊙\Sigma \mu -\mu^{2}⊙diag(\Sigma )\right)$$

$$cov(x,z)=2\left(\mu^{T}\Sigma -\mu ⊙diag(\Sigma )\right)$$

Combining these vectorized formulas for the moments of the variables $z_{i}$ with the vectorized formula for γ, we can efficiently compute the approximation to the first moment γ of the projected normal distribution.

### B.2 Vectorized formula for $E\left[{yy}^{T}\right]$ approximation

To obtain a vectorized formula for the second moment matrix $E\left[{yy}^{T}\right]$ of the projected normal, we can first define the matrices $\bar{N}\in R^{n\times n}$ and $cov(N,d)\in R^{n\times n}$, with elements ${\bar{N}}_{ij}={\overset{‾}{n}}_{ij}$ and $cov(N,d{)}_{ij}=cov\left(n_{ij},d\right)$. Then we can define the following vectorized formula for the second moment matrix (compare to Equation 4 in the main text):

$$E\left[{yy}^{T}\right]\approx \frac{\bar{N}}{\overset{‾}{d}}⊙\left(1-\frac{cov(N,d)}{\bar{N}\overset{‾}{d}}+\frac{1\;\cdot \;var(d)}{{\overset{‾}{d}}^{2}}\right)$$

where the divisions between vectors are element-wise.

Then, like for the first moment γ, the moments of the auxiliary variable $\bar{N}$ and cov(N,d) can be computed using efficient vectorized formulas. For example, consider the formula for ${\overset{‾}{n}}_{ij}$ in the main text. It is clear that

$$\bar{N}=\Sigma +\mu \mu^{T}$$

Then, consider the formula for $cov\left(n_{ij},d\right)$ in the main text. It is easy to show that the first term $2tr\left(\Sigma A^{ij}\Sigma \right)$ is equal to $2\Sigma_{i:}\Sigma_{:j}$, where $\Sigma_{i:}$ is the i’th row of Σ and $\Sigma_{:j}$ is the j’th column. Thus, the matrix product 2ΣΣ has the corresponding element $2\Sigma_{i:}\Sigma_{:j}$ at each position (i,j). Applying similar algebraic manipulations to the second term, we obtain the following vectorized formula for cov(N,d):

$$cov(N,d)=2\left(\Sigma \Sigma +\mu \mu^{T}\Sigma +\Sigma \mu \mu^{T}\right)$$

Then, using the vectorized formulas for $\overset{-}{N}$ and cov(N,d), we can compute the second moment matrix $E\left[{yy}^{T}\right]$ of the projected normal distribution using the vectorized formula above.

## Appendix C Exact moments of $𝒫𝒩\left(\mu ,\sigma^{2}I\right)$

### C.1 Exact solution for γ

For the case where $\Sigma =I\sigma^{2}$, an exact closed-form formula can be derived for γ. To obtain the closed-form formula for γ, we first note that because of the symmetry of x around μ, γ will be parallel to μ. Therefore, we only need to solve for the expected value of the projection of y onto the direction of μ

(C5)
$$E\left[y^{T}\cdot \frac{\mu }{\left\|\mu \right\|}\right]=E\left[\frac{x^{T}\mu }{\left\|x\|\|\mu \right\|}\right]=\;\frac{1}{{\left(2\pi \sigma^{2}\right)}^{\frac{n}{2}}}\int_{R^{n}}\;e^{-\frac{1}{2\sigma^{2}}(x-\mu {)}^{T}(x-\mu )}\frac{x^{T}\mu }{\left\|x\|\|\mu \right\|}dx=\;\frac{1}{{\left(2\pi \sigma^{2}\right)}^{\frac{n}{2}}}e^{-\frac{\mu^{2}}{2\sigma^{2}}}\int_{R^{n}}\;e^{-\frac{r^{2}}{2\sigma^{2}}}e^{\frac{r\|\mu \|}{\sigma^{2}}cos(\theta )}cos(\theta )dx=\;\frac{1}{{\left(2\pi \sigma^{2}\right)}^{\frac{n}{2}}}e^{-\frac{\mu^{2}}{2\sigma^{2}}}\int_{0}^{\infty }\;dr\int_{0}^{\pi }\;r{\;A}_{n-2}(r\;sin(\theta ))\;e^{-\frac{r^{2}}{2\sigma^{2}}}e^{\frac{r\|\mu \|}{\sigma^{2}}cos(\theta )}cos(\theta )d\theta$$

where

$$A_{n-2}(r\;sin(\theta ))=\frac{2\pi^{(n-1)/2}}{\Gamma \left(\frac{n-1}{2}\right)}r^{n-2}{sin}^{n-2}(\theta )$$

is the area of the n-2 sphere of radius rsin(θ), and θ is the angle between x and μ. In the first step above, we expanded the term $\left(x-\mu {)}^{T}(x-\mu \right)$, we used the identity $\frac{x\cdot \mu }{\left\|x\|\|\mu \right\|}=cos(\theta )$, where θ is the angle between x and μ, and denoted the norm of ‖x‖ as r, refering to the radius from the origin to the point x. In the second step, we decomposed the integral into polar coordinates using the angle θ between x and μ. The angular components other than θ are encompassed by the area $A_{n-2}(r\;sin(\theta ))$. This is because for a fixed r and θ, all the points defined by the remaining angular directions form an n-2 sphere of radius rsin(θ) where all the points have the same probability (because of symmetry around μ), and the integrand does not depend on these directions. Thus, the integral over the remaining angular directions is equal to the area of the n-2 sphere of radius rsin(θ).

Grouping all θ-dependent terms in the integral and substituting cos(θ)=z, ${sin}^{n-2}(\theta )={\left(1-z^{2}\right)}^{\frac{n-2}{2}}$ and $d\theta =-{\left(1-z^{2}\right)}^{-\frac{1}{2}}dz$, we can evaluate the integral with respect to θ using an integral representation of the hypergeometric function F01

∫1-1-zer‖μ‖σ2z1-z2n-22-12dz=π2Γn-12Γn2+1‖μ‖rσ2F01n2+1;‖μ‖r2σ22

Using this integral representation, moving outside of the integral the terms that do not depend on r, and simplifying, we obtain the following

ExTμ‖x‖‖μ‖=12σ2n21Γn2+1‖μ‖σ2e-‖μ‖22σ2∫0∞rne-r22σ2F01n2+1;‖μ‖r2σ22dr

Next, substituting $h=\frac{r^{2}}{2\sigma^{2}}$ and $dr=\frac{\sigma^{2}}{\sqrt{2h}}dh$, simplifying, and using the integral representation F11(a;b;h)Γ(a)=∫0∞e-tta-1F01(b;ht)dt we obtain

(C6)
EyT⋅μ‖μ‖=12σ2121Γn2+1‖μ‖e-‖μ‖22σ2×∫0∞hn2-12e-hF01n2+1;μ22σ2hdh=12σ212Γn+12Γn2+1‖μ‖e-‖μ‖22σ2×F11n+12;n2+1;‖μ‖22σ2=12σ212Γn+12Γn2+1‖μ‖F1112;n2+1;-‖μ‖22σ2

where in the last step we used Kummer's transformation F11(a;b;m)=emF11(b-a;b;-m). Thus, multiplying the result of Equation C6 by μ/‖μ‖, we obtain the closed-form formula for γ :

(C7)
γ=12σ212Γn+12Γn2+1×F1112;n2+1;-‖μ‖22σ2μ

### C.2 Exact solution for $E\left[yy^{T}\right]$

As mentioned in Section A, each element of $E\left[{yy}^{T}\right]$ is the expectation of a ratio of quadratic forms in random variables $E\left[n_{ij}/d\right]$ where $n_{ij}=x^{T}A^{ij}x$ and $d=x^{T}x$, where x~𝒩(μ,Σ). For the special case where $\Sigma =\sigma^{2}I$, the expectation of this ratio of quadratic forms has an exact solution (Smith 1993, 1996)

(C8)
ExTMxxTx=tr(M)nF111;n2+1;-‖μ‖22σ2+μTMμn+2F111;n2+2;-‖μ‖22σ2

Thus, the value of $E[y_{i}y_{j}^{T}]$ can be obtained by substituting $M=A^{ij}$ in Equation C8, with $A^{ij}$ being the matrix defined in Section A. Noting that $tr\left(A^{ij}\right)$ is 1 when i=j and 0 otherwise, and that $\mu^{T}A^{ij}\mu =\mu_{i}\mu_{j}$, it is easy to verify the following matrix formula

(C9)
EyyT=1nF111;n2+1;-‖μ‖22σ2I+1n+2F111;n2+2;-‖μ‖22σ2μμT
